# Comparison of multiple imaging modalities for measuring orifice diameter and selecting occluder size in patients undergoing left atrial appendage closure

**DOI:** 10.1002/clc.23869

**Published:** 2022-06-08

**Authors:** Kandi Zhang, Jing Zhou, Tiantian Zhang, Zongqi Zhang, Shanliang Jin, Qing He, Junfeng Zhang

**Affiliations:** ^1^ Department of Cardiology Ninth People's Hospital Affiliated to Shanghai Jiao Tong University School of Medicine Shanghai China; ^2^ Department of Anesthesiology Ninth People's Hospital Affiliated to Shanghai Jiao Tong University School of Medicine Shanghai China

**Keywords:** 3D reconstruction, atrial fibrillation, left atrial appendage occlusion, occluder size

## Abstract

**Background:**

Left atrial appendage (LAA) closure (LAAC) can safely and effectively prevent stroke events caused by atrial fibrillation. However, the structure of the LAA is highly variable among individuals, and the optimal method for obtaining measurements remains unknown.

**Hypothesis:**

We aimed to study the accuracy of left atrial computed tomography angiography (CTA), three‐dimensional (3D) reconstruction using CTA, two‐dimensional transesophageal echocardiography (2D‐TEE), and digital subtraction angiography (DSA) for measuring the diameter of the LAA and compare their value for selecting occluder size.

**Methods:**

We retrospectively evaluated data for 148 patients with nonvalvular atrial fibrillation who underwent successful LAAC. CTA and 2D‐TEE of the left atrium and pulmonary vein were performed before LAAC. We performed 3D reconstruction of the left atrium and LAA using Mimics and 3‐matics software. DSA of the LAA was performed during surgery.

**Results:**

Values measured via CTA 3D reconstruction were significantly higher than those measured using other methods. DSA‐measured values were significantly lower than those measured via CTA and CTA 3D reconstruction. Occluder size was positively correlated with LAA orifice diameter. The differences between occluder size and DSA, 2D‐TEE, CTA, CTA 3D reconstruction measurements were 4.96 ± 2.58, 4.64 ± 2.50, 4.04 ± 1.37, and 2.92 ± 1.38 mm, respectively. Intraclass correlation coefficients for these methods were −.067, .006, .241, and .519, respectively.

**Conclusion:**

CTA 3D reconstruction provides the best correlation and consistency between the measured LAA orifice diameter and occluder size. Adding 2–4 mm to the maximum LAA orifice diameter based on 3D‐CTA may aid in selecting the appropriate WATCHMAN device.

## 1 INTRODUCTION

Atrial fibrillation is a common symptom of rapid arrhythmia in middle‐aged and older individuals, and its incidence increases with age. During atrial fibrillation, a thrombus tends to form in the left atrial appendage (LAA), originating from this area in 90% of cardiogenic thrombus. A recent study reported that this rate increases to almost 100% in patients with nonvalvular atrial fibrillation (NVAF).^1^ Emboli that develop following thrombus shedding represent the main cause of death and disability in patients with atrial fibrillation.^2^

Left atrial appendage closure (LAAC) has been recommended to reduce the risk of stroke following atrial fibrillation. LAAC aims to prevent thrombus formation in the LAA during atrial fibrillation via an occluder implanted at the LAA orifice, thereby reducing the risk of long‐term disability or death caused by thromboembolism. The size and morphology of the LAA vary significantly among patients. Preoperative computed tomography angiography (CTA) three‐dimensional (3D) reconstruction, two‐dimensional transesophageal echocardiography (2D‐TEE), and intraoperative digital subtraction angiography (DSA) are often used to evaluate the size, depth, and axial direction of the LAA and provide adequate guidance for operations. However, values have differed substantially among studies. The early prospective WATCHMAN trials suggested that an average of 1.8 occluders were required for each occlusion operation, reported successful occlusion in 82% of cases.^3^ One of the most common reasons for reselecting the occluder in this study was that the originally selected device was too small, indicating that selecting occluder sizer based on the size of LAA on DSA or TEE may not be the most accurate method. This study aimed to compare the value of different imaging modalities for measuring the LAA orifice diameter and selecting occluder size.

## 2 MATERIALS AND METHODS

### 2.1 General information

We retrospectively collected data for 148 patients (83 men, 65 women; mean age, 72.70 ± 8.30 years) with NVAF who had undergone successful LAA closure surgery at Shanghai Ninth People's Hospital from January 2020 to December 2020. Sixty‐six patients (44.6%) had persistent atrial fibrillation, while 82 (55.4%) had paroxysmal atrial fibrillation. The mean CHA_2_DS_2_‐VASc and hypertension stroke‐bleeding labile INRs elderly drugs or alcohol (HAS‐BLED) scores were 4.45 ± 1.51 and 2.92 ± 1.06, respectively. A total of 57 patients (38.5%) underwent a single procedure involving radiofrequency ablation and LAAC. Twenty‐three patients (15.5%) had a medical history of hemorrhage (such as gastrointestinal bleeding and cerebral hemorrhage) and bleeding tendency. The detailed clinical characteristics of the included patients are listed in Table 1.

**Table 1:** Baseline characteristics of the study population.

	*N* = 148
Age, year	72.70 ± 8.30
Female	65 (43.92%)
BMI, kg/m^2^	25.17 ± 2.19
Paroxysmal AF	76 (51.35%)
CHA_2_DS_2_‐VASc score	4.45 ± 1.51
HAS‐BLED score	2.92 ± 1.06
Hypertension	121 (81.76%)
Diabetes	36 (24.32%)
Vascular disease	61 (41.22%)
Chronic heart disease	19 (12.84%)
Stroke or TIA	66 (44.59%)

Abbreviations: BMI, body mass index; TIA, transient ischemic attacks.

LAA morphology was described as the cauliflower type in 95 patients (64.2%), the chicken‐wing type in 23 patients (15.5%), the windsock type in 15 patients (10.1%), the cactus type in four patients (2.7%), and other in 11 patients (7.4%). WATCHMAN occluders were used in all cases (Boston Science Company). The release of occluders satisfied the “position, anchor, size, and seal” principle and the residual shunt was ≤5mm. The immediate compression ratio was 22.25 ± 3.33 mm as measured via TEE. There were nine cases of residual shunting, which were distributed as follows: 1 mm, *n* = 1 case; 2 mm, *n* = 5 cases; 3 mm, *n* = 3 cases. Thus, all cases met standards for occluder release.

All patients were followed‐up via telephone. Three patients died within 6 months after the operation, including two patients with cancer and one patient with possible pulmonary embolism. A total of 113 patients completed the 2D‐TEE follow‐up within 3 months after the operation, two of whom experienced device‐related thrombus (DRT) formation. Follow‐up revealed residual shunting in four cases (all ≤3 mm). Residual shunting appeared immediately after the operation in three of the four cases, while no immediate residual shunting was observed in the remaining case.

### 2.2 Examination methods

#### 2.2.1 DSA

**Figure 1:**
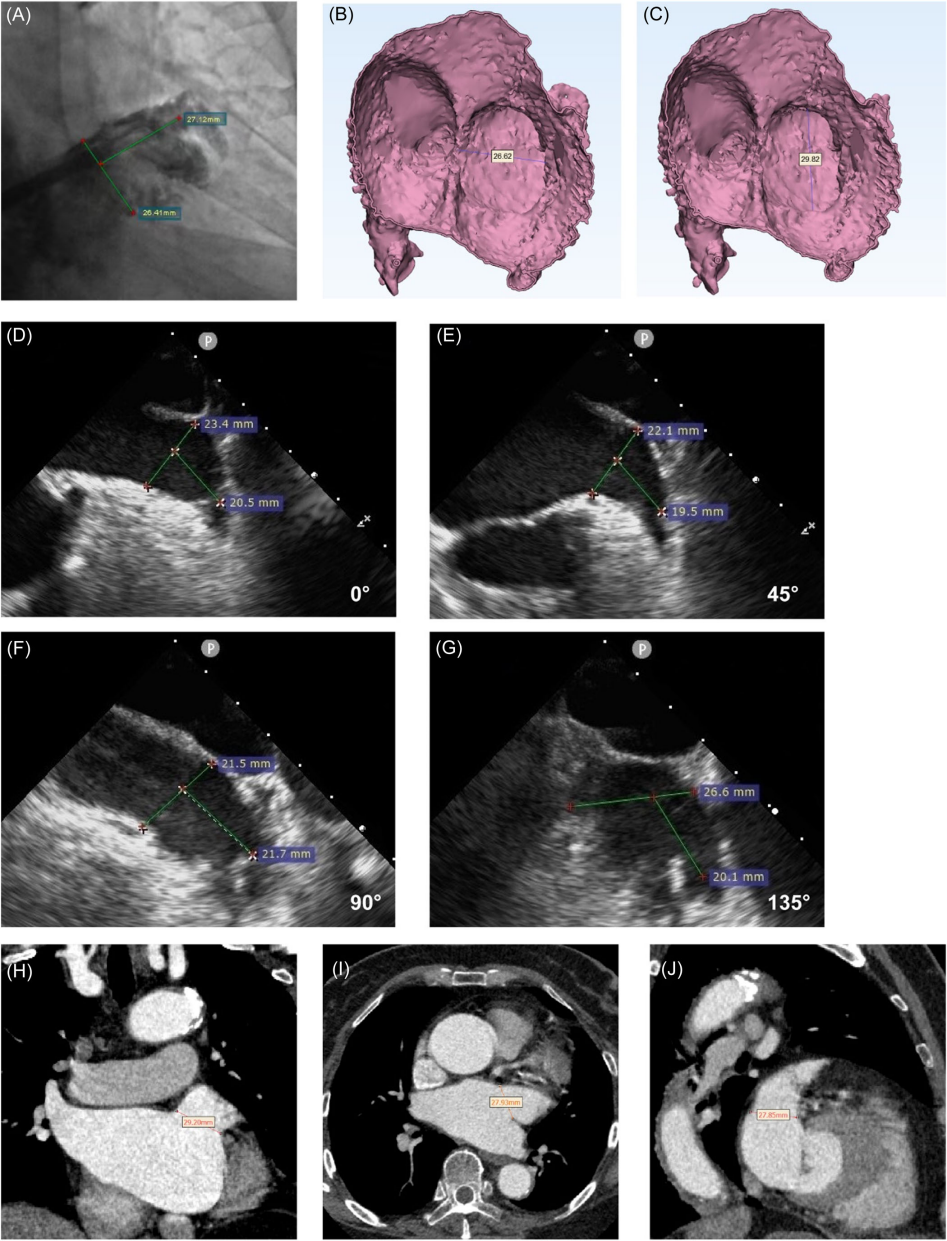
LAA orifice diameter as measured by different imaging methods. (A) Digital subtraction angiography at the 30° right anterior oblique position +20° caudal position to measure the LAA orifice diameter. (B, C) LAA orifice diameter was measured by 3D reconstruction of CTA using Mimics software. (D–G) LAA orifice diameter was measured in four sections at 0°, 45°, 90°, and 135°, respectively, by transesophageal echocardiography. (H–J) LAA orifice diameter was measured by multiplanar reconstruction with CT angiography in different positions (coronal, sagittal, and transverse positions). CT, computed tomography; CTA, computed tomography angiography; LAA, left atrial appendage.

With the patients under intravenous anesthesia, LAA angiography was performed with a sheath and pigtail catheter in the working position (30° right anterior oblique + 20° caudal). The maximum LAA orifice diameter was measured using the LAA image obtained at this position (Figure 1A).

#### 2.2.2 2D‐TEE

A Philips IE33 color Doppler echocardiography system (Philips) was used for the 2D‐TEE examination. The X7‐2t probe was used at a mid‐esophageal depth of 30–35 cm. LAA morphology, orifice diameter, thrombus formation in the LAA, and spontaneous development grade were observed from 0°, 45°, 90°, and 135° views. The LAA orifice diameter was recorded as the maximum distance between the left superior pulmonary vein and the origin of the circumflex branch in different sections (Figure 1D–F). The 2D‐TEE images without color Doppler were acquired at 46 Hz, while those with the color flow were acquired at 12 Hz.

#### 2.2.3 CTA

Before surgery, all patients underwent CTA examination of the left atrium using a 64‐slice SOMATOM definition flash dual‐source computed tomography (CT) scanner (Siemens). The temporal resolution was 330 ms and, the detector collimation was 64 × 0.6 mm. Iodixanol diluted with normal saline was injected through the elbow vein at the rate of 5 ml/s as the contrast agent. Following injection, scans were performed after a delay of 60 s to ensure adequate circulation of the contrast agent. Images of the left atrium were obtained in the end‐diastolic phase. The LAA orifice diameter was measured in the coronal, sagittal, and transverse positions, and the maximum value was recorded as the CTA measurement (Figure 1H–J). All images were analyzed by two independent, experienced radiologists. The average value of the measurement was taken as the result.

#### 2.2.4 CTA 3D reconstruction using Mimics software

The image data obtained from left atrial CTA was imported into Materializer's Interactive Medical Image Control System (Mimics) (Materialise) software in DICOM format. Soft tissue was represented in grayscale, following which the left atrium, left ventricle, right atrium, right ventricle, aorta, and pulmonary artery were selected separately and added into the CT heart segmentation. The calculated masks were copied into 3‐Matics 12.0 (Materialise) software, and the hollow function (distance set to 1 mm; smallest detail set to 0.1 mm) was used to completely reconstruct the left atrium, including the structural model of the LAA and pulmonary vein. The trim function was used to fully expose the section of LAA orifice. The maximum diameter was recorded as the LAA orifice diameter (Figure 1B,C).

### 2.3 Statistical analyses

Statistical analysis was performed by SPSS 22.0 (IBM). Measurement data conforming to a normal distribution were described as the mean and standard deviation, while those not conforming to a normal distribution were described as the median and quartile. The Shapiro–Wilk test was used to test for normality. Normally distributed measurement data were compared between two groups using *t*‐tests and among multiple groups using analyses of variance. The SNK‐q test was selected for pairwise comparisons in cases of statistical significance. The Wilcoxon Mann–Whitney *U* rank‐sum test was used to compare nonnormally distributed data between two groups, while the Kruskal–Wallis *H* test was used for comparisons among multiple groups. The Bonferroni method was used for pairwise comparisons in cases of statistical significance. Pearson correlation coefficients were used to analyze the correlation between two variables whose measurement data conformed to a normal distribution, while Spearman rank correlation coefficients were used when data from one or both groups did not conform to a normal distribution. Intraclass correlation coefficients (ICCs) and the Bland–Altman method were used to analyze intergroup consistency. The level of statistical significance was set at *p* < .05.

## 3 RESULTS

### 3.1 Comparison of LAA orifice diameter measured using different imaging methods

The LAA orifice diameter measured via CTA 3D reconstruction (25.20 ± 2.85 mm) was larger than that measured via intraoperative DSA, 2D‐TEE, or CTA (23.15 ± 2.79, 23.48 ± 2.82, 24.07 ± 2.57 mm, respectively; *p* < .05). The results are shown in Table 2.

**Table 2:** LAA orifice diameter as measured by different imaging methods.

Imaging methods	Mean ± standard deviation (mm)	Median (quartiles) (mm)
DSA	23.15 ± 2.79^a^	23.08 (21.31, 25.33)^a^
2D‐TEE	23.48 ± 2.82^a^	23.61 (21.82, 25.40)^a^
CTA	24.07 ± 2.57^a^, ^b^, ^c^	24.23 (22.42, 25.00)^a^, ^b^, ^c^
CTA 3D reconstruction	25.20 ± 2.85^a^, ^b^, ^c^, ^d^	25.27 (23.30, 27.23)^a^, ^b^, ^c^, ^d^
Occluder size	28.12 ± 3.04	27.00 (27.00, 30.00)

*Note*: Since the 2D‐TEE, CTA, and occlude size did not meet the normal distribution, the Kruskal–Wallis *H* test was used and the Bonferroni method was used for pairwise comparison.

Abbreviations: 2D‐TEE, two‐dimensional transesophageal echocardiography; 3D, three‐dimensional; CTA, computed tomography angiography; DSA: digital subtraction angiography; LAA, left atrial appendage.

^a^ Indicates that compared with occluder size, *p* < .05.

^b^ Indicates that compared with DSA, *p* < .05.

^c^ Indicates that compared with ultrasound, *p* < .05.

^d^ Indicates that compared with CTA, *p* < .05.

**Figure 2:**
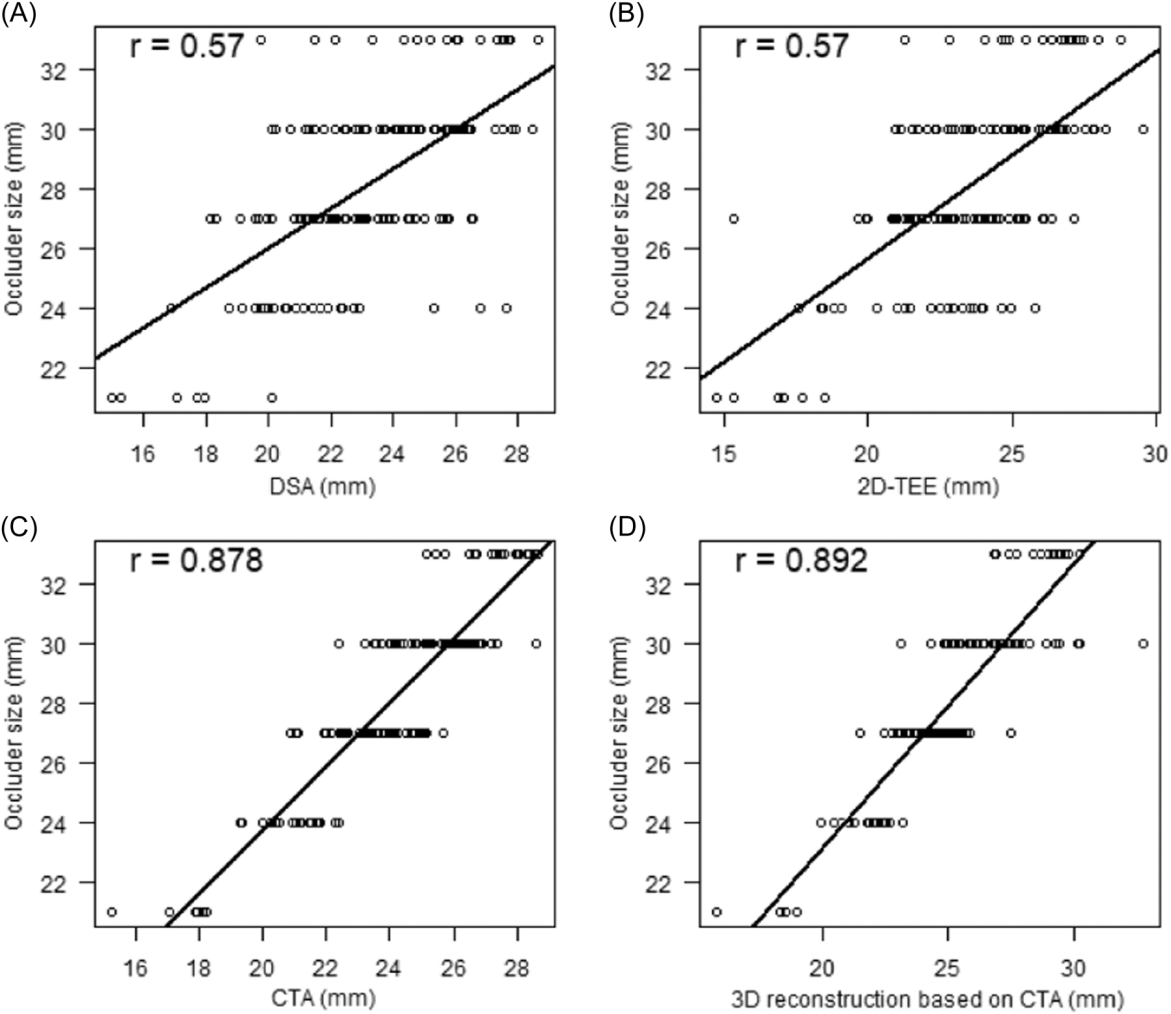
Correlation between LAA orifice diameter as measured by different imaging methods and occluder size. 2D‐TEE, two‐dimensional transesophageal echocardiography; 3D, three‐dimensional; CTA, computed tomography angiography; DSA, digital subtraction angiography; LAA, left atrial appendage.

The occluder size was positively correlated with LAA orifice diameter as measured by DSA, 2D‐TEE, CTA, and CTA 3D reconstruction (*r *= .57, .57, .88, .89, all *p* < .001) (Table 3 and Figure 2).

**Table 3:** Correlation between LAA orifice diameter as measured by different imaging methods and occluder size.

Imaging methods	*r*	*W*	*p*
DSA and occluder size	.57	232252.10	<.001
2D‐TEE and occluder size	.57	232162.30	<.001
CTA and occluder size	.88	66013.57	<.001
CTA 3D reconstruction and occluder size	.89	58381.65	<.001

*Note*: Since the 2D‐TEE, CTA, and occlude size did not meet the normal distribution, the Spearman rank correlation analysis and Wilcoxon signed‐rank test were used.

Abbreviations: 2D‐TEE, two‐dimensional transesophageal echocardiography; 3D, three‐dimensional; CTA, computed tomography angiography; DSA, digital subtraction angiography; LAA, left atrial appendage.

### 3.2 Consistency between LAA orifice diameter as measured using different imaging methods and occluder size

**Figure 3:**
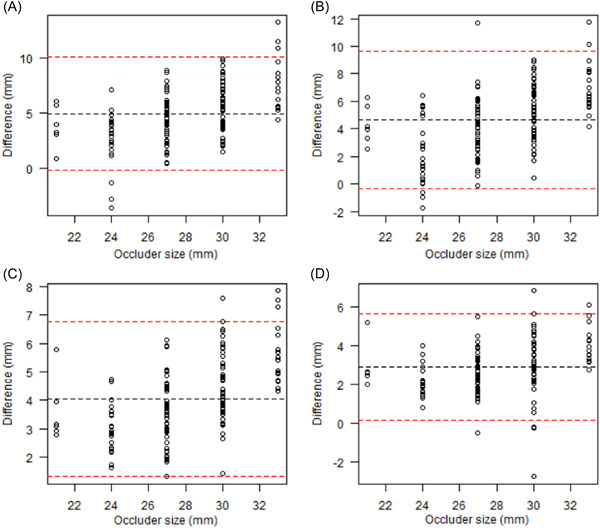
Consistency between LAA orifice diameter as measured by different imaging methods and occluder size. (Bland–Altman) (A) DSA, (B) 2D‐TEE, (C) CTA, (D) CTA 3D reconstruction. 2D‐TEE, two‐dimensional transesophageal echocardiography; 3D, three‐dimensional; CTA, computed tomography angiography; DSA, digital subtraction angiography; LAA, left atrial appendage.

Bland–Altman plots and ICCs were used to evaluate the consistency between the reference value and LAA orifice diameter measured using each of the four methods. The consistency limit was narrowest for CTA (1.31, 6.78 mm), followed by CTA 3D reconstruction (0.16, 5.67 mm), while it was widest for (−0.25, 10.13 mm). The difference from the reference value was 4.96 ± 2.58 mm (95% confidence interval [CI]: −0.14, 10.07 mm) for DSA, 4.64 ± 2.50 mm (95% CI: −0.30, 9.57 mm) for 2D‐TEE, 4.04 ± 1.37 mm (95% CI: 1.34, 6.74 mm) for CTA, and 2.92 ± 1.38 mm (95% CI: 0.19, 5.64 mm) for CTA 3D reconstruction (Figure 3).

The ICCs for occluder size and LAA orifice diameter were largest for CTA 3D reconstruction (0.52), followed by CTA (0.24) (*p* < .05). The ICCs of occluder size and LAA orifice diameter measured using DSA and 2D‐TEE were −0.07 and 0.01, respectively; however, the difference was not statistically significant (Table 4).

**Table 4:** Consistency between LAA orifice diameter as measured by different imaging methods and occluder size.

Imaging method	ICC	*p*
DSA	−0.067	.793
2D‐TEE	0.006	.469
CTA	0.241	.001
CTA 3D reconstruction	0.519	<.001

Abbreviations: 2D‐TEE, two‐dimensional transesophageal echocardiography; 3D, three‐dimensional; CTA, computed tomography angiography; DSA, digital subtraction angiography; ICC, intraclass correlation coefficient; LAA, left atrial appendage.

## 4 DISCUSSION

Atrial fibrillation is one of the most common arrhythmias in older adults, and the risks of stroke and heart failure are significantly higher among patients with atrial fibrillation than among those without. The LAA is a remnant structure of the left atrium from early embryonic development, and it is located between the anterior and posterior walls of the left atrium. Abundant comb‐like muscle structures are present on the medial surface. When atrial fibrillation occurs, the rate of blood flow to the atria decreases significantly, which can lead to thrombus formation in the LAA. In recent years, with the optimization of the LAA occluder and the maturation of surgical technology, an increasing number of clinical studies have shown that LAAC can isolate the LAA from the left atrium via placement of an occluder, safely and effectively preventing stroke events caused by atrial fibrillation.^3^, ^4^

The size and effective depth of the LAA have important reference values that allow for the selection of the appropriate occluder type and size. However, the structure of the LAA is highly variable among individuals, and the optimal method for obtaining these measurements remains to be determined.

In the DSA method, contrast agent is injected into the LAA during operation to make the blood flow in the LAA visible under X‐ray. In the TEE method, the ultrasonic probe is placed into the esophagus to allow for the exploration of the deep structures of the heart from the rear to the front. Compared with transthoracic echocardiography (TTE), TEE can avoid the interference of obesity, lung gas, and bone and provide images of higher quality. Preoperative TEE combined with intraoperative DSA is often used to guide the surgery. The EHRA/EAPCI Expert Consensus Statement on Catheter‐based Left Atrial Appendage Occlusion—An Update^5^ recommends that LAAC be performed under general anesthesia and TEE. TEE can aid the surgeon in evaluating the thrombus in the left atrium and LAA, enable them to puncture the atrial septum, and allow for assessment of LAA structure.

Although TEE is currently recommended as the preferred imaging examination for preoperative evaluation, intraoperative guidance, and postoperative follow‐up after LAAC,^6^ the shape of the LAA is irregular and highly variable. Thus, it is sometimes difficult to obtain or omit the true maximum section, resulting in a smaller measured value. In addition, the surgeon's experience and equipment will also affect measurement accuracy during TEE. Similarly, when DSA is performed in the working position (30° right anterior oblique + 20° caudal), it is difficult to obtain the tangent direction corresponding to the length of the LAA orifice in the projection position. The LAA orifice may overlap with the left pulmonary vein crest or the left atrium, meaning that the LAA orifice may not be fully exposed. These issues may lead to the selection of an inappropriate occluder. Occluders that are too small can easily shift or fall off, while those that are too large can compress or damage the surrounding tissue. In addition, a small compression ratio can lead to residual shunting, impairing the occlusion effect and increasing the risk of DRT. Studies of the WATCHMAN occluder have also suggested that too small or too large a compression ratio can increase the incidence of residual shunting.^7^ Furthermore, the following disadvantages have been highlighted: (1) The results of TEE are greatly affected by the operator's experience and equipment‐related errors, such as artifact interference; (2) TEE is a semi‐invasive examination that can be highly uncomfortable for patients; (3) patients may exhibit poor tolerance to TEE under local anesthesia.

Multislice spiral CT involves volumetric scanning under continuous rotation. Most clinical research studies have used coronary CTA or delayed enhanced left atrial CTA for LAA. CTA has high spatial resolution and is not affected by body position or operator experience, allowing for objective and accurate measurements with high repeatability and consistency. It is also advantageous in that it is simple, noninvasive, and associated with good patient tolerance, making it suitable for preoperative evaluation and postoperative follow‐up of patients undergoing LAAC. Nonetheless, researchers have begun to focus on 3D reconstruction of LAA images to obtain more accurate measurements.^8^ The powerful 3D reconstruction and postprocessing functions of Mimics and 3‐matic software can convert CTA data into a 3D model of the cardiac structure, improve visualization of the LAA shape, and provide technical support for evaluating the size and shape of the LAA as well as its relationship with surrounding tissues. At the same time, 3D‐CTA is also advantageous in that it utilizes the original CTA data. Moreover, assessing the shape of the LAA using 3D‐CTA preoperatively may help to reduce radiation exposure and the dosage of contrast medium during the operation while increasing the success rate for occluder selection. A small‐sample single‐center study in China reported on the use of the Mimics simulated 3D imaging system for visualizing the LAA in patients with atrial fibrillation before surgery, demonstrating that the system provided the necessary information and allowed for the success of LAAC.^9^

One study indicated that LAA values measured using DSA, 2D‐TEE, and coronary CTA differed significantly.^10^ Coronary CTA measurements deviated from TEE measurements by 3 mm, while 2D‐TEE measurements deviated from DSA measurements by 3 mm. Chow et al.^7^ found that the selection of the occluder size based on cardiac multislice spiral CT is more accurate than that based on traditional 2D‐TEE. Studies have also suggested that, when compared with 2D‐TEE, coronary CTA improves the accuracy of occlusion device selection and the success rate of surgery. Our study showed that the size of the implanted occluder was closer to the LAA orifice diameter measured using 3D‐CTA. A recent single‐center study also reported that technological improvements in 2D‐TEE have reduced the difference between 2D‐TEE and 3D CTA group, although the latter was associated with better accuracy in terms of first device selection.^11^

Our study has some limitations, including its single‐center retrospective design, highlighting the need for a prospective multicenter study with a larger sample size for further verification. The occluders used in this study were all “internal plug” WATCHMAN occluders, while cases with "external cover" occluders were excluded. Thus, our conclusions may be not applicable to LAAC procedures utilizing “external cover” occluders such as ACP, LACbes, and LAmbre occluders. Further, we only utilized 2D‐TEE in the current study. However, real‐time 3D‐TEE exhibits greater functionality and better image quality, which may provide more structural information regarding the LAA.

Specifically, 3D‐TEE overcomes the insufficient spatial resolution of 2D‐TEE. It can slice the image from any angle or plane stereoscopically and intuitively without the interference of spontaneous development, making the hit highly applicable for real‐time evaluation of LAA morphology. Particularly, compared with 2D‐TEE, 3D‐TEE can better distinguish thrombi and comb muscle/trabecula^12^; allow for dynamic observation of systolic and diastolic movement as well as volume changes in the LAA^13^; and ensure better consistency between the measured value and the size of the occluder.^11^

Compared with DSA and 2D‐TEE, CTA 3D reconstruction is advantageous in that it can be performed preoperatively and has been associated with good patient tolerance. It is an excellent, accurate, and intuitive technical means for the preoperative evaluation of LAA. The results of the present study show that the LAA orifice diameter values obtained via CTA 3D reconstruction were greater than those by obtained via DSA and 2D‐TEE. There was no significant difference between the values measured using DSA and 2D‐TEE. Our findings also indicated that the LAA orifice diameter measured using CTA 3D reconstruction was closer to the size of the final implanted occluder, exhibiting better consistency and suggesting the greater value of this method for selecting the occluder size. Adding 2–4 mm to the value of 3D‐CTA may represent a useful reference for selecting the size of the WATCHMAN occluder during operation.

## CONFLICT OF INTEREST

The authors declare no conflicts of interest.

## Data Availability

The datasets used or analyzed during the current study are available from the corresponding author on reasonable request.
